# Adaptive Policy Switching for Multi-Agent ASVs in Multi-Objective Aquatic Cleaning Environments

**DOI:** 10.3390/s26020427

**Published:** 2026-01-09

**Authors:** Dame Seck, Samuel Yanes-Luis, Manuel Perales-Esteve, Sergio Toral Marín, Daniel Gutiérrez-Reina

**Affiliations:** 1 Department of Electronic Engineering, University of Sevilla, 41004 Seville, Spain; dseck@us.es (D.S.); mperales@us.es (M.P.-E.); storal@us.es (S.T.M.); 2 Department of Electronic Technology, University of Sevilla, 41004 Seville, Spain; syanes@us.es

**Keywords:** multi-task multi-agent deep reinforcement learning, multi-objective optimization, autonomous surface vehicles, environmental monitoring, partially observable markov games

## Abstract

Plastic pollution in aquatic environments is a major ecological problem requiring scalable autonomous solutions for cleanup. This study addresses the coordination of multiple Autonomous Surface Vehicles by formulating the problem as a Partially Observable Markov Game and decoupling the mission into two tasks: exploration to maximize coverage and cleaning to collect trash. These tasks share navigation requirements but present conflicting goals, motivating a multi-objective learning approach. The proposed multi-agent deep reinforcement learning framework involves the utilisation of the same Multitask Deep Q-network shared by all the agents, with a convolutional backbone and two heads, one dedicated to exploration and the other to cleaning. Parameter sharing and egocentric state design leverages agent homogeneity and enable experience aggregation across tasks. An adaptive mechanism governs task switching, combining task-specific rewards with a weighted aggregation and selecting tasks via a reward-greedy strategy. This enables the construction of Pareto fronts capturing non-dominated solutions. The framework demonstrates improvements over fixed-phase approaches, improving hypervolume and uniformity metrics by 14% and 300%, respectively. It also adapts to diverse initial trash distributions, providing decision-makers with a portfolio of effective and adaptive strategies for autonomous plastic cleanup.

## 1. Introduction

Macro-plastic (plastic > 25 mm) persists in aquatic environments, threatening ecosystems through entanglement, ingestion, and the trophic transfer of toxic additives [1,2]. Despite advances in mitigation and cleanup technologies using Autonomous Surface/Unmanned Surface Vehicles (ASVs/USVs) [3,4,5,6], a primary challenge in plastic cleanup missions remains the uncertainty of trash distribution before deployment [7,8,9]. Plastic contamination is highly dynamic—driven by water currents, wind, and human activity—which undermines static trajectory planning and limits the efficiency of deterministic control or classical optimization approaches. Recent advances in Deep Reinforcement Learning (DRL) have demonstrated the capacity to handle such uncertainty by enabling adaptive, data-driven decision-making. Canonical methods such as Deep Q-Learning (DQL) [10] and its extensions like Rainbow DQN [11] have shown strong performance in high-dimensional control tasks, including pollution monitoring [12,13,14] and trash collection [9]. To extend these capabilities to coordinated multi-robot systems, we model the cleanup mission as a Partially Observable Markov Game (POMG) [15], which naturally captures both the uncertainty of trash locations and the interactions among multiple ASVs operating simultaneously. The POMG generalizes the classical Markov Decision Process (MDP) to multi-agent scenarios where each vehicle senses only partial observations of the environment, reflecting realistic sensing and communication constraints. POMG thereby provides a mathematical foundation for Multi-Agent DRL (MADRL), which leverages parameter sharing to improve scalability and coordination across ASV fleets [16,17].

To manage mission complexity, we decouple the mission into two distinct tasks: exploration, where agents aim to cover the map to localize trash, and cleaning, where they focus on trash collection. Multi-task learning has been used in this context to jointly optimize policies for both objectives using Multi-task DQN (MDQN) architectures [7,8,18]. Unlike prior studies that relied on fixed-phase strategies, conducting exploration first and cleaning afterward [8], we treat task allocation as a dynamic decision variable. Specifically, a binary switch variable ν∈{0,1} enables each agent to adaptively select between exploratory and cleaning policies. Therefore, agents can shift toward cleaning when nearby plastic concentrations are high or return to exploration when the local area becomes depleted. This flexible task allocation introduces an inherent trade-off: excessive exploration reduces cleaning time, while insufficient exploration risks leaving undetected debris.

The use of Multi-Objective DRL (MO-DRL) has gained increasing attention for resolving trade-offs between conflicting objectives. Foundational studies such as [19,20,21] developed techniques for learning Pareto-optimal policies through scalarization and preference-conditioned networks. Building on these ideas, by training across varying allocations of ν, we construct Pareto-optimal fronts that capture the trade-offs between exploration coverage and cleaning efficiency. Recent studies have shown that, beyond uncertainty alone, aquatic environments fundamentally violate many of the assumptions underlying standard MO-DRL methods [22]. Even single-objective DRL has been shown to struggle with aquatic navigation due to non-stationary dynamics, degraded sensing and violations of the Markov property [22]. These challenges are further amplified in the multi-objective setting, where sparse, delayed and constrained objectives make reward design and scalarization more difficult, limiting the applicability of existing MO-DRL approaches to realistic aquatic missions. To support adaptive decision-making, we also introduce a reward-greedy policy selection mechanism inspired by submodular optimization principles [23,24]. This mechanism evaluates all possible task assignments across the fleet and selects the one that maximizes a weighted combination of exploration and cleaning performance. By systematically varying the weights, the framework reconstructs a diverse Pareto front, providing decision-makers with a portfolio of strategies tailored to different mission priorities. In summary, we adopt a Multi-Task Multi-Agent DRL (MT-MADRL) framework solved via multi-objective optimization. Our contributions are twofold: (i) a multi-task multi-agent DRL framework for ASV-based plastic cleanup that decouples exploration and cleaning while enabling adaptive task switching; (ii) a multi-objective optimization formulation that leverages reward decomposition and Pareto front construction to balance exploration and cleaning under diverse mission requirements.

The rest of this paper continues as follows: Section 2 introduces the environmental framework, including the scenario characteristics, system dynamics, and the methodology used throughout the study. It also describes the DRL formulation, the policy network architecture and training procedure, the state and reward definitions, and the proposed policy-selection mechanism. Section 3 details the simulation setup and presents the experimental results, including a comparison between the proposed method and existing approaches in the literature. Finally, Section 4 provides concluding remarks and outlines directions for future research.

## 2. Materials and Methods

### 2.1. Problem Formulation

We formulate the plastic cleanup mission as a POMG [25] involving *N* homogeneous ASVs operating in a discretized aquatic environment. The POMG framework naturally captures the agents’ limited perception and the interactions among multiple ASVs. Although agents share observations through centralized communication to construct a collective trash map $\hat{Y}$, this map represents only the last known state of the environment and may diverge from the true trash distribution *Y* due to continuous trash dynamics and sensing delays. Furthermore, regions outside the perception radius of all agents remain unobserved. The overall mission objective is to remove trash as efficiently as possible, which implicitly requires effective coordination, safe navigation, and energy-aware trajectories. In order to locate and remove trash items, it is necessary to explore the environment. Rather than considering this as an implicit part of the cleaning process, we have decided to decouple the learning process into two dedicated policies: an exploration policy, where agents aim to maximize coverage of the environment, and a cleaning policy, where agents collect as much identified trash as possible once locations are known. Both tasks share underlying requirements such as efficient navigation and obstacle avoidance, yet they present conflicting objectives: allocating more effort to exploration improves trash localization but reduces the time available for collection, while prioritizing cleaning without sufficient exploration leads to incomplete recovery. This inherent tension motivates a multi-objective learning framework.

Formally, let π denote a policy and let *D* be the number of objectives. Each policy produces a vector of performance metrics $X_{\pi }\in R^{D}$ across episodes due to stochastic trash distributions. The expected performance is $f_{\pi }=\left(f_{\pi }^{1},\ldots ,f_{\pi }^{D}\right)$, where $f_{\pi }^{i}=E\left[X_{\pi }^{i}\right]$. In our case, D=2, with $f_{\pi }^{1}$ and $f_{\pi }^{2}$ representing expected exploration and cleaning performance, respectively. A policy $\pi^{*}$ is Pareto-optimal if there is no other policy $\pi^{\prime }$ such that:

(1) $$f_{\pi^{\prime }}^{i}\geq f_{\pi^{*}}^{i}\;\forall i\in \left\{1,2\right\},\;\mathrm{with}\;\mathrm{strict}\;\mathrm{inequality}\;\mathrm{for}\;\mathrm{at}\;\mathrm{least}\;\mathrm{one}\;\mathrm{objective}.$$

The aim of this work is thus twofold: (i) to learn specialized exploration and cleaning policies, and (ii) to construct a Pareto front of solutions that captures the optimal trade-offs between the two objectives.

### 2.2. Scenario and Vehicle Properties

**Figure 1:**
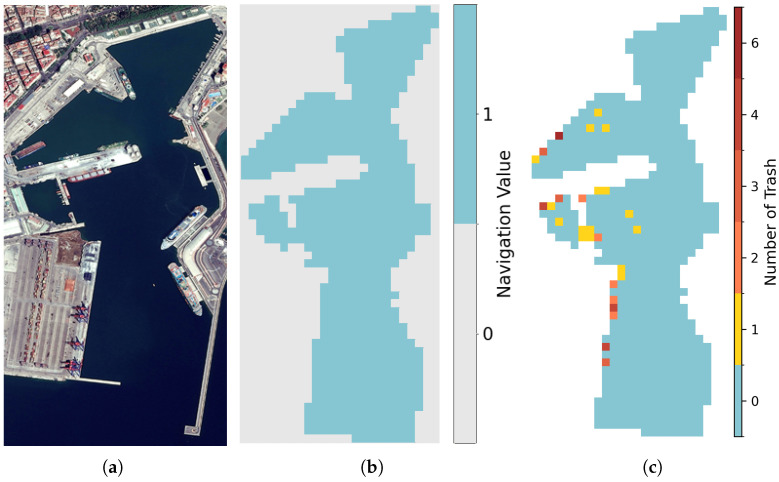
Examples of the (**a**) Malaga Port map in Spain (**b**) transformed into a matrix M[i,j]∈{0,1} and (**c**) an example of the matrix *Y* (trash distribution) at a certain step.

Let G=(V,E) denote a grid-based map of size H×W structured as a connected graph, where

$$V=\{v_{i,j}|1\leq i\leq H,\;1\leq j\leq W\}$$

is the set of nodes, with each node $v_{i,j}\in V$ corresponding to a location in the grid. The set of edges connecting adjacent nodes is denoted as E⊆V×V, indicating possible movements between neighboring nodes. Each node connects to its eight immediate neighbors, making the grid eight-connected. Nodes with fewer than eight connections represent the presence of obstacles. The graph *G* can also be represented as a binary matrix M[i,j]∈{0,1} of size H×W (see Figure 1b):

(2) $$M\left[i,j\right]=\left\{\begin{array}{cc} 1 & \mathrm{if}\;\mathrm{the}\;\mathrm{node}\;v_{i,j}\;\mathrm{is}\;\mathrm{navigable}\\ 0 & \mathrm{otherwise} \end{array}\right.$$

**Figure 2:**
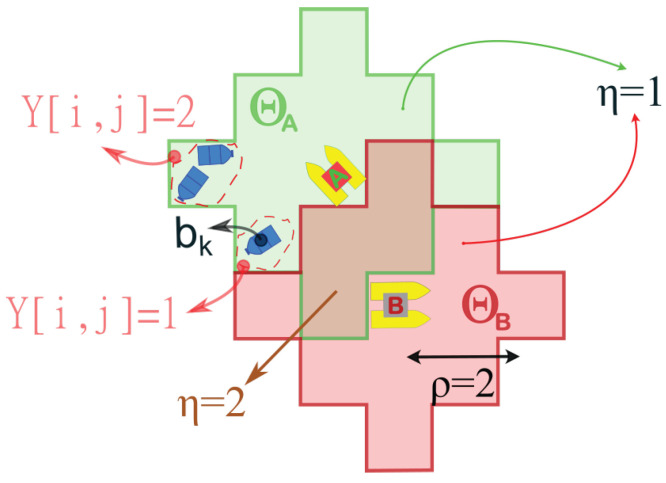
Illustration of the perception areas $\Theta (p_{n})$ for two vehicles (A and B) in a discretized environment with a vision radius ρ=2. The function η(v,P) represents the number of agents covering each cell, while Y[i,j] indicates the number of trash items located within that cell after discretization.

Let *N* denote the number of ASVs. The fleet position in *G* at time *t* is given by $P=\{p_{n}^{t}|n=1,2,\ldots ,N\}$, where $p_{n}^{t}=v_{i,j}\in V$ is the position of vehicle *n*. ASVs move synchronously in one of the eight cardinal directions (N, E, S, W, NE, SE, NW, SW), with each simulation step corresponding to one discrete movement. Actions leading to non-navigable nodes (M[i,j]=0) are masked to ensure valid transitions. When multiple ASVs attempt to occupy the same node, conflicts are resolved via a consensus algorithm [14], preventing inter-vehicle collisions. Energy constraints are directly modeled as a finite movement budget corresponding to battery capacity. Each agent’s episode ends after exceeding this distance budget, making policies implicitly energy-aware. Each ASV is also equipped with a camera. The perception area of vehicle *n* is defined as:

$$\Theta \left(p_{n}\right)=\{v\in V|∥v-p_{n}∥<\rho \}$$

where ρ is the vision radius and $∥v-p_{n}∥$ denotes Euclidean distance (see Figure 2).

### 2.3. Trash Dynamics and Recollection

Let $B=\{b_{k}=\left(x_{k},y_{k}\right)|k=1,\ldots ,K\}$ denote the set of trash positions, where *K* is the total number of items and $\left(x_{k},y_{k}\right)$ are continuous coordinates. At the beginning of each episode, trash is generated in 1–10 clusters, with items sampled from a multivariate normal distribution. The dynamics of each item follow:

$$b_{k}^{t+1}=b_{k}^{t}+\Delta t\left(w_{wind}\cdot \nu_{wind}+w_{rand}\cdot \nu_{rand}\right),$$

where $\nu_{wind}∼U\left(-1,1\right)$ is sampled once per episode, and $\nu_{rand}∼U\left(-1,1\right)$ is sampled independently at each step. Weights are fixed at $w_{wind}=0.1$ and $w_{rand}=0.01$. Trash items are discretized into a matrix $Y\in N^{H\times W}$ (see Figure 1c and Figure 2), where:

$$Y\left[i,j\right]=|\{b_{k}\in B|\left(x_{k},y_{k}\right)\in \mathrm{Area}\left(i,j\right)\}|,$$

with Area(i,j) denoting the region corresponding to cell (i,j). Each agent maintains an estimated trash map $\hat{Y}\left[i,j\right]$, initialized to zero and updated whenever $v_{i,j}\in \Theta \left(p_{n}\right)$:

$$\hat{Y}\left[i,j\right]\leftarrow Y\left[i,j\right].$$

Agents hare their observations via centralized communication, mitigating some limitations of partial observability but still subject to the uncertainty of trash dynamics. Inter-agent communication delays and robustness to disturbances are not explicitly modeled and are left for future work. However, the learning architecture itself is fleet-size independent, as agents rely on relative state information rather than a fixed team structure. Nevertheless, this property does not imply computational scalability of the task-allocation mechanism. When an ASV occupies $p_{n}=v_{i,j}$, it collects all trash at that node, with collection capacity assumed unlimited. The number of items collected is:

$$C\left(p_{n},Y\right)=Y\left[i,j\right]|v_{i,j}=p_{n}.$$

### 2.4. Deep Reinforcement Learning Framework

Reinforcement Learning (RL) [26] addresses sequential decision-making by modeling the interaction between agents and the environment as a MDP (S,A,T,R,γ). At each step *t*, an agent observes a state $s_{t}$, selects an action $a_{t}$, transitions to $s_{t+1}∼T\left(s_{t+1}|s_{t},a_{t}\right)$, and receives a reward $r_{t}$. The objective is to learn a policy π that maximizes the expected cumulative reward. Q-Learning estimates the action-value function $Q_{\pi }\left(s,a\right)$, the expected return of taking action *a* in state *s* under π. Deep Q-Networks (DQNs) [10] approximate Q(s,a) with a neural network parameterized by θ, updated via:

(3) $$Q\left(s,a;\theta \right)\leftarrow Q\left(s,a;\theta \right)+\alpha (r+\gamma \max_{a^{\prime }}Q\left(s^{\prime },a^{\prime };\theta \right)-Q\left(s,a;\theta \right)),$$

where α is the learning rate. Stability and performance are enhanced through mechanisms such as Experience Replay, Target Networks, Double DQN [27], and the Dueling Architecture [28].

MADRL generalizes DQL to a cooperative setting with *N* homogeneous agents acting in a shared environment, formalized as a POMG (N,S,A,O,T,R,γ). Each agent n∈N receives a local observation $o_{n}^{t}$, selects an action $a_{n}^{t}$, and receives a reward $R_{n}$, while the environment evolves according to the joint action $a^{t}=\left(a_{1}^{t},\ldots ,a_{N}^{t}\right)$. Each agent aims to maximize its long-term return:

(4) $$J_{\pi_{n},\pi_{-n}}\left(s\right)=E_{a^{t}∼\pi }\left[\sum_{t=0}^{\infty }\gamma^{t}R_{n}^{t}\left(s_{t},a^{t},s_{t+1}\right)|s=s_{t}\right],$$

where −n denotes all agents except *n*. In our cooperative ASV cleanup scenario, parameter sharing [29] is applied: all agents use the same policy network, allowing experiences to be aggregated, reducing computational cost, and ensuring scalability. To improve credit assignment, agents are rewarded based on their individual contributions to the team’s performance [30].

To further improve learning, the mission is formulated as a multi-task problem with two tasks: *exploration*, which aims to maximize coverage and locate trash, and *cleaning*, which focuses on collecting the identified trash. While the state and action spaces are shared, transition dynamics $T^{\left(z\right)}$ and rewards $R^{\left(z\right)}$ differ per task z∈Z, producing a vectorized reward signal. Following [18], Multi-task Deep Q-Networks (MDQN) employ separate Q-heads per task while sharing the underlying feature representation. This allows knowledge gained in exploration, such as efficient navigation and obstacle avoidance, to transfer to cleaning, improving overall mission performance.

### 2.5. Policy Network Training

#### 2.5.1. Phase Construction

Following [7], the mission is divided into two phases to handle initially unknown trash locations: *Exploration* and *Cleaning*. The transition between phases is controlled by a variable ν∈{0,1}, representing the probability of selecting an exploration action:

$$\pi_{\nu }\left(s\right)=\left\{\begin{array}{cc} \underset{a}{\arg\max}Q_{e}\left(s,a\right), & \mathrm{with}\;\mathrm{probability}\;\nu \\ \underset{a}{\arg\max}Q_{c}\left(s,a\right), & \mathrm{with}\;\mathrm{probability}\;1-\nu \end{array}\right.$$

where $Q_{e}$ and $Q_{c}$ are the Q-functions for exploration and cleaning, respectively. In the *Exploration* phase, ν=1 at every step, so all actions are chosen according to the exploratory policy $Q_{e}$, with the primary goal of maximizing map coverage and discovering trash locations. In the *Cleaning* phase, ν=0, so actions are fully governed by the cleaning policy $Q_{c}$, aiming to recollect the detected trash efficiently.

#### 2.5.2. Action Space

Each autonomous surface vehicle operates in a discrete action space

$$A=\left\{a_{1},\ldots ,a_{8}\right\}=\left\{N,S,E,W,\mathrm{NE},\mathrm{NW},\mathrm{SE},\mathrm{SW}\right\},$$

corresponding to the eight possible movement directions on the discretized grid. Actions that would result in collisions with obstacles or boundary violations are masked and excluded from execution, ensuring safe navigation within the environment.

#### 2.5.3. States

The state representation for agent *n* is egocentric and composed of three H×W min-max normalized channels:

- *Trash model*: $\hat{Y}\left[i,j\right]$, shared among all agents, representing detected trash positions and capturing partial observability.
- *Agent trail position*: records the agent’s detection history over the past 10 steps, with current detection cells at 1 and past steps decaying by 0.1 per step, down to 0.1.
- *Position of other agents*: the union of detection masks Θ of all other agents, with cells inside the masks set to 1, providing awareness of fleet distribution.

Although this work assumes a homogeneous fleet for clarity, heterogeneity in autonomy, sensors, or speed can be handled through the shared contamination model and agent-specific state channels. Position remains a universal attribute, and the contamination model can be extended to reflect sensor variability.

#### 2.5.4. Rewards

At each step *t*, each agent *n* receives a vector of two rewards $R_{t,n}=\left[R_{e,n},R_{c,n}\right]$, corresponding to exploration and cleaning objectives.

The Exploration Reward ($R_{e}$) encourages discovery of new areas while penalizing inactivity and redundancy:

$$R_{e,n}=r_{vr,n}-p_{ip,n}-p_{rp,n}$$

- *Visit reward* $r_{vr,n}$: proportional to newly discovered cells, shared among overlapping agents:
  $$r_{vr,n}=\frac{\sum U^{t}\left(\Theta \left(p_{n}\right)\right)-U^{t-1}\left(\Theta \left(p_{n}\right)\right)}{\eta (v,P)}$$
  where $\eta \left(v,P\right)=|\{p_{n}\in P|∥v-p_{n}∥<\rho \}|$ counts overlapping coverage.
- *Inactivity penalty* $p_{ip,n}$: penalizes consecutive steps without discovering new areas:
  $$p_{ip,n}=\frac{1}{\delta }\cdot \xi ,\;\xi =\left\{\begin{array}{cc} \xi +1, & \mathrm{if}\;\mathrm{no}\;\mathrm{new}\;\mathrm{cells}\;\mathrm{discovered}\\ 0, & \mathrm{otherwise} \end{array}\right.$$
  where ξ accumulates the number of consecutive steps without discovering new cells, and δ scales this penalty to control its impact.
- *Redundancy penalty* $p_{rp,n}$: penalizes overlap with areas explored by self or other agents in the last τ steps:
  $$p_{rp,n}=\frac{1}{|\Theta (p_{n}^{t})|\cdot \tau }\sum_{t^{\prime }=t-\tau }^{t-1}\sum_{m\neq n}\left|\Theta \left(p_{n}^{t}\right)\cap \Theta \left(p_{m}^{t^{\prime }}\right)\right|$$

The Cleaning Reward ($R_{c}$) encourages trash collection and efficient movement towards trash:

$$R_{c,n}=r_{tcr,n}+r_{mu,n}+r_{d,n}-p_{t,n}$$

- *Trash collection reward* $r_{tcr,n}=C\left(p_{n},Y\right)$: rewards collecting trash at current position.
- *Distance reward* $r_{d,n}$: promotes movement towards nearby trash using inverse Dijkstra distances:
  $$r_{d,n}=\frac{1}{|\Theta \left(p_{n}^{t}\right)|\cdot \eta \left(v,P\right)}\sum_{v\in \Theta (p_{n})}\sum_{b\in B}\frac{1}{d(v,b)}$$
- *Model update reward* $r_{mu,n}={\hat{Y}}^{t}\left(\Theta \left(p_{n}\right)\right)-{\hat{Y}}^{t-1}\left(\Theta \left(p_{n}\right)\right)$: encourages adaptation to dynamic trash movement.
- *Time penalty* $p_{t,n}=1$: discourages unnecessary delays.

#### 2.5.5. Multi-Task Policy Network

The network containing the two task policies is trained following the Randomized ν-Sampling for Pareto Front Approximation Algorithm as in [8]. All agents share a single MDQN-based policy network composed of a dense Convolutional Neural Network (CNN) with two dueling Q-heads, one for exploration and one for cleaning. Parameter sharing is employed because the agents are homogeneous in both observations and actions, while the egocentric state formulation allows the policy architecture to generalize to arbitrary fleet sizes without structural modification. Each Q-head is trained independently using its respective task-specific rewards through DQL. The utilisation of shared layers facilitates the aggregation of experiences derived from both tasks, thereby reducing computational cost and enhancing sample efficiency. In order to ensure diverse learning across the exploration—cleaning trade-off, the duration of the exploration phase is sampled at the beginning of each episode. This duration determines when ν transitions from 1 (pure exploration) to 0 (pure cleaning) over the course of the mission. By varying the phase length across episodes, the network learns policies covering the full spectrum of task allocations. At the end of training, the network contains two fully trained policies, exploration $\pi_{e}$ and cleaning $\pi_{c}$.

### 2.6. Reward-Greedy Policy Selection and Pareto Front Construction

**Figure 3:**
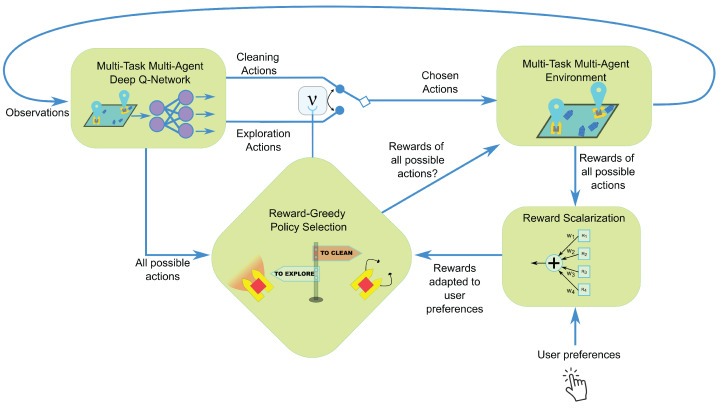
Diagram of the proposed algorithm. At each step, the environment provides an observation, and all possible task combinations (corresponding to different ν configurations) are simulated across the fleet. The resulting rewards are computed and scalarized according to user-defined preferences. The Reward-Greedy Policy Selection mechanism then identifies the task combination yielding the highest reward. The selected actions are executed, and the environment advances to produce the next observation.

To enhance adaptive decision-making, we introduce a Reward-Greedy Policy Selection (RGPS) mechanism. At each decision step, RGPS evaluates all possible task allocations across the fleet, specifically whether each ASV should perform exploration or cleaning. Given two possible tasks per vehicle, this results in $2^{N}$ possible task combinations for a fleet of *N* ASVs. Each combination is simulated to compute its expected scalarized reward, which aggregates exploration and cleaning objectives according to a given weighting of objectives. The combination yielding the highest weighted reward is selected, and each agent executes the corresponding action from its designated policy (exploration or cleaning). This process defines the fleet’s joint behavior at every step. The RGPS mechanism follows the principle of greedy submodular optimization, which is well-established for near-optimal decision-making in adaptive and stochastic settings [23,24]. In submodular systems, greedy maximization provides strong theoretical guarantees for solution quality under uncertainty—properties that align well with our partially observable and dynamically changing aquatic environment. Since the marginal benefit of additional exploratory actions typically diminishes as more trash is detected, the problem exhibits approximately submodular behavior. The Reward-Greedy method is simple to implement and it does not require additional learning. It explicitly considers all feasible task assignments, ensuring near-optimal short-term decisions based on the chosen reward weighting. Moreover, it leverages the separate training of exploration and cleaning policies, allowing agents to specialize while maintaining coordination. Figure 3 shows the diagram that summarizes our proposed algorithm. The RGPS task allocation mechanism is executed on a centralized server (base station) that receives observations from all ASVs, computes the optimal task assignment, and transmits individual action commands back to each vehicle. This centralized architecture leverages existing shore-based infrastructure commonly available in harbor and coastal operations.

Weighting Method: To balance exploration and cleaning, we introduce a weighting coefficient w∈[0,1] that combines the two objectives into a single scalar optimization function. This methods allow the user to specify preferences, which may be articulated in terms of goals or the relative importance of different objectives. To mitigate bias introduced by any single scalarization, several complementary weighting strategies are employed, including linear, multiplicative, power-based, and exponential formulations. Each scalarization explores different regions of the Pareto front, and their combination significantly improves coverage and diversity. Following the most common general scalarization methods for multi-objective optimization [31], with $\left(w_{1},w_{2}\right)=\left(w,\;1-w\right)$ and $\left(R_{1},R_{2}\right)=\left(R_{\mathrm{clean}},R_{\mathrm{explore}}\right)$, the combined objective can be expressed using either:

- Weighted Sum (WS): The most common approach to multi-objective optimization is the weighted sum method, which is essentially a convex combination of the two rewards, it suffers from the disadvantage of not being able to find a diverse set solutions if the Pareto front is non-convex.
  (5) $$R_{\mathrm{total}}\;=\;\sum_{i=1}^{2}w_{i}R_{i}$$
- Weighted Power (WP): In this method, each reward is raised to a power α before applying the weight. This allows for emphasizing certain objectives non-linearly and can be tuned via the parameter α:
  (6) $$R_{\mathrm{total}}\;=\;\sum_{i=1}^{2}w_{i}R_{i}^{\alpha }$$
- Weighted Product of Powers (WPOP): This multiplicative approach combines the rewards by taking each reward to the power of its respective weight and then multiplying them. It is useful for emphasizing that all objectives should be high, as a low value in any objective strongly reduces the total scalarized reward:
  (7) $$R_{\mathrm{total}}\;=\;\prod_{i=1}^{2}R_{i}^{w_{i}}$$
- Exponential Weighted Criterion (EWC): In response to the inability of the weighted sum method to capture points on non-convex portions of the Pareto optimal surface, Ref. [32] propose the exponential weighted criterion, the performance of the method depends on the value of *p* and usually a large value of *p* is needed.
  (8) $$R_{\mathrm{total}}=\sum_{i=1}^{k}\left(e^{pw_{i}}-1\right)e^{pR_{i}}$$

These formulations provide a flexible trade-off between the two competing objectives, enabling systematic exploration of the Pareto front. The RGPS mechanism uses this weighted reward to evaluate all possible policy assignments, selecting the combination that maximizes $R_{\mathrm{total}}$ at each step. By repeating this process across different weightings *w* and multiple episodes, we obtain a set of policies whose expected performance in exploration and cleaning spans the trade-off space. Plotting the expected $R_{\mathrm{explore}}$ versus $R_{\mathrm{clean}}$ for these policies forms a Pareto front, representing the set of non-dominated strategies. Decision-makers can then select policies from the Pareto front according to mission priorities, ensuring an explicit balance between maximizing trash collection and achieving full coverage of the environment.

Novelty and Relationship to Prior Work: This work builds upon our previous study [8] in which we introduced a multi-task DQN framework for ASV fleet clean-up involving a fixed-phase decomposition between exploration and clean-up. In that approach, all agents transitioned synchronously between phases at a predefined timestep. In order to enable a fair comparison, we have retained the same environment, state representation, reward formulation and multi-task DQN architecture as in [8]. The key novelty of the present work lies in the task coordination mechanism. RGPS is an adaptive, per-agent task selection method that operates at every decision step, replacing the global, time-based phase switch. RGPS enables agents to dynamically specialise based on current environmental conditions, allowing for asynchronous and fine-grained task allocation across the fleet. This results in emergent coordinated behaviour without the need for additional learning modules or explicit communication, representing a significant departure from the fixed-phase strategy employed in [8].

## 3. Results

The proposed algorithm has been implemented in Python 3 (https://www.python.org/, accessed on 5 January 2025), using the PyTorch (https://pytorch.org/, accessed on 5 January 2025) library for policy optimization. The Gym (https://gymnasium.farama.org/, accessed on 5 January 2025) has been used to simulate the environment, NumPy (https://numpy.org/, accessed on 5 January 2025) and SciPy (https://scipy.org/, accessed on 5 January 2025) libraries for numerical and matrix operations. Pareto Fronts were constructed using Pymoo (https://pymoo.org/, accessed on 5 January 2025). The algorithm’s code and results are accessible in a GitHub repository (https://github.com/dsdiop/MultiProfilePlasticCleaningProblem, accessed on 5 January 2025). All experiments and simulations were run on an Intel Xeon Gold 5220R CPU operating at 2.20 GHz with 187 GB of RAM. Additionally, a NVIDIA GeForce RTX 3090 GPU with 24 GB of VRAM was employed to accelerate training.

### 3.1. Simulation Settings

The environment consists of four ASVs, each equipped with a detection radius of 2 nodes, a movement budget of 200 units per episode, and a movement step size of 1 node. The ASVs start from four fixed initial positions. Horizontal and vertical movements incur a cost of 1 unit, while diagonal movements cost $\sqrt{2}\approx 1.414$ units.

Table 1 summarizes the environment and training parameters. These values are intentionally kept consistent with our previous work [8] in order to isolate the impact of the proposed adaptive task allocation mechanism, rather than confounding the comparison with changes in environment or learning hyperparameters.

**Greedy Execution:** The agents are evaluated over 100 episodes using the Reward-Greedy strategy. For each episode, a weight vector is sampled from a uniform distribution w∼U(0,1), where one weight w∈[0,1] is drawn and the other is set to 1−w, ensuring that the sum of weights equals one. After completing the 100 episodes for a given weight vector, the mean values of the exploration metric (PMV) and the cleaning metric (PTC) are computed. This process is repeated for 100 different weight combinations. Finally, the Pareto Front is constructed from the resulting set of 100 (PTC,PMV) metric pairs. The entire procedure is performed separately for each weight-based scalarization method. The path planner that is going to choose the action once the decision to explore or clean is taken by the RGPS agent, is trained as in [8].

### 3.2. Metrics and Hyperparameter Sensitivity Analysis

The performance is evaluated fof each policy using a set of objective metrics that quantify exploration efficiency and cleaning effectiveness.

- Percentage of the Map Visited (PMV): It is the exploration objective to be maximized, and it measures the percentage of the map that has been visited during the mission. It is defined as the proportion of the total navigable area that has been visited at least once by any ASV. A higher PMV value indicates a more comprehensive exploration, ensuring that the agents gather sufficient information about the environment’s structure, obstacles, and trash distribution, which is crucial for the subsequent cleaning phase. Mathematically, PMV is given by:
  (9) $$PMV=\frac{\sum_{i}\sum_{j}U^{T}\left[i,j\right]}{\sum_{i}\sum_{j}M\left[i,j\right]}\cdot 100$$
- Percentage of Trash Cleaned (PTC): It is the cleaning objective to be maximized, and it measures the proportion of trash that has been effectively eliminated from the environment, relative to the total amount of trash present at the beginning of the episode (*K*). Optimizing PTC requires efficient path planning and coordination among agents to maximize trash collection before exceeding their distance limits. PTC is mathematically defined as:
  (10) $$PTC=\frac{\sum_{t}^{T}\sum_{p_{n}\in P}C\left(p_{n},Y^{t}\right)}{K}\cdot 100$$

To characterize the trade-offs between these objectives, we compute Pareto front metrics that capture the diversity, extension and quality of the resulting solutions.

- Consecutive Spacing: The spacing metric measures the variance of distances between consecutive solutions along the Pareto front, sorted by the first objective $f_{1}$. It captures gaps along the front better than nearest-neighbor spacing in 2D.
  For a sorted Pareto front $Y_{N}=\left\{y_{1},y_{2},\ldots ,y_{N}\right\}$, the consecutive distances are
  (11) $$d_{i}=∥y_{i+1}-y_{i}∥,\;i=1,\ldots ,N-1,$$
  and the spacing metric is defined as the standard deviation of these distances:
  (12) $$S=\sqrt{\frac{1}{N-2}\sum_{i=1}^{N-1}{\left(d_{i}-\bar{d}\right)}^{2}},$$
  where
  (13) $$\bar{d}=\frac{1}{N-1}\sum_{i=1}^{N-1}d_{i}.$$
  A lower *S* indicates more uniform spacing along the front.
- Zitzler’s $M_{3}$ Metric: The $M_{3}$ metric measures the extent of the Pareto front along all objectives. For a Pareto front $Y_{N}=\left\{y_{1},y_{2},\ldots ,y_{N}\right\}$ in *m* objectives, let
  (14) $$ZR_{i}=\max_{1\leq j\leq N}y_{j}^{\left(i\right)}-\min_{1\leq j\leq N}y_{j}^{\left(i\right)},\;i=1,\ldots ,m,$$
  be the range of the front along the *i*-th objective. Then the $M_{3}$ metric is defined as
  (15) $$ZM_{3}=\sqrt{\sum_{i=1}^{m}ZR_{i}}.$$
  A higher $M_{3}$ indicates a larger extent of the front across all objectives.
- Hypervolume (HV): The hypervolume quantifies the size of the objective space dominated by the Pareto Front (PF) with respect to a reference point $r=(r_{1},r_{2})$. For discrete non-dominated points sorted by $f_{1}$ (e.g., PTC), the hypervolume can be approximated as the sum of rectangular areas between consecutive PF points:
  (16) $$HV=\sum_{i=1}^{N}\left(r_{1}-f_{1}^{\left(i\right)}\right)\;\left(f_{2}^{\left(i\right)}-f_{2}^{\left(i-1\right)}\right)$$
  where $f_{1}^{\left(i\right)}$ and $f_{2}^{\left(i\right)}$ denote the objective values of the *i*-th non-dominated solution in the sorted PF, and $f_{2}^{\left(0\right)}=r_{2}$ corresponds to the reference point. A higher HV indicates a larger and more dominant Pareto region, reflecting both convergence and diversity.

**Figure 4:**
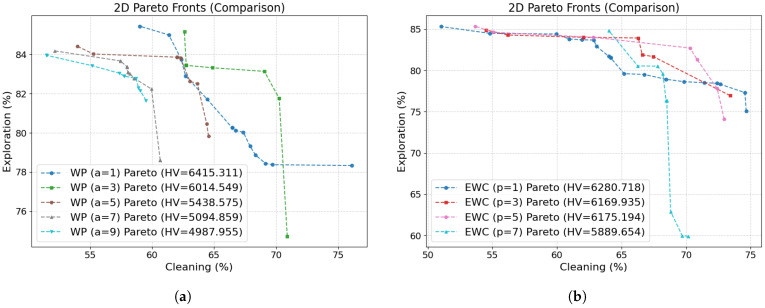
Hyperparameter sensitivity analysis of scalarization methods. Comparison of 2D Pareto fronts obtained by varying the exponent parameters: (**a**) *a* in the Weighted Power method and (**b**) *p* in the Exponential Weighted Criterion method.

Hyperparameter Sensitivity Analysis: To assess the robustness of the proposed framework with respect to scalarization hyperparameters, we conducted a sensitivity analysis on the parameters governing the non-linear scalarization methods. Specifically, we evaluated the effect of the exponent *a* in the Weighted Power (WP) method and the parameter *p* in the Exponential Weighted Criterion (EWC) method on the resulting Pareto fronts. For WP, we considered a∈{1,3,5,7,9}, while for EWC we evaluated p∈{1,3,5,7}. The analysis focuses on the Hypervolume metric as the primary indicator of multiobjective performance, complemented by the M3 and Spacing metrics. The results show that, although absolute metric values vary with the choice of parameters, the qualitative behavior of the framework remains consistent across the tested ranges. Moderate parameter values yield balanced Pareto coverage, whereas more extreme values increasingly bias the scalarization toward specific objectives. Based on this analysis, we select a=3 for WP and p=1 for EWC in the experiments reported in this work. Figure 4a shows that increasing the WP exponent *a* progressively compresses the Pareto front and reduces coverage of balanced trade-offs between cleaning and exploration. We therefore select a=3 as a moderate setting that preserves a well-spread Pareto front without the strong bias and loss of diversity observed for larger values. Moreover, we select p=1 for EWC as it yields the highest hypervolume (see Figure 4b) while avoiding the increased bias and Pareto front distortion observed for larger values of *p*.

### 3.3. Scalarization Results

**Figure 5:**
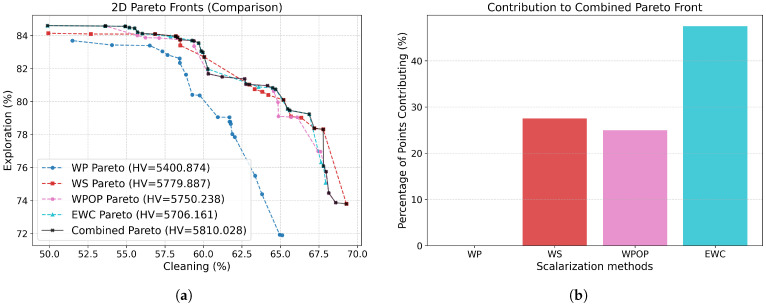
Results of the scalarization methods in Málaga Port scenario. (**a**) Pareto fronts obtained using each scalarization method across 100 different weight configurations, along with the resulting combined Pareto front that aggregates all non-dominated solutions. (**b**) Contribution of each scalarization method to the combined Pareto front.

As illustrated in Figure 5, the Pareto fronts obtained for each scalarization method under the Reward-Greedy Policy Selection (RGPS) framework are presented, in conjunction with the combined Pareto set which integrates all scalarizations to exploit the complete information available. The corresponding performance metrics are summarised in Table 2. Each Pareto point corresponds to the mean performance over 100 independent evaluation episodes under fixed trained policies, rather than to individual trajectories.

It is evident that the WP method exhibits significantly poorer performance in comparison to the alternative approaches. The Wilcoxon signed-rank test, conducted between Pareto fronts, revealed that the remaining methods do not exhibit statistically significant differences (Table 3). The WS approach achieves the highest hypervolume; however, it is known that it is limited to convex regions of the Pareto front. In contrast, the WPOP and EWC methods, both non-linear scalarizations, achieve slightly lower hypervolume and Pareto extension ($M_{3}$) values but yield improved Spacing due to their ability to explore non-convex regions. Furthermore, due to the convex-constrained nature of WS, numerous weight combinations tend to converge towards the boundary of the front, thereby leading to elevated $M_{3}$ values.

When all scalarization results are combined, the best overall metrics are achieved, along with an increased number of Pareto-optimal points. Figure 5b illustrates the contribution of each scalarization method to the combined Pareto front. Out of 40 total Pareto points, EWC contributes the majority (47.50%), followed by WS (27.50%) and WPOP (25.00%), while WP does not contribute. This highlights the importance of employing multiple scalarization techniques of distinct natures (linear, non-linear, additive, multiplicative, and exponential) to achieve a well-distributed and comprehensive Pareto front. The combined front represents our proposed contribution and will serve as the basis for comparison against alternative algorithms from the literature.

#### 3.3.1. Comparison

For benchmarking against existing approaches, we adopt the methodology introduced in [8], hereafter referred to as the *Fixed-Phase Pareto Set* (FP2S). A predefined set of phase transition configurations, denoted as the Phase Duration Set (PDS), is used to generate possible phase durations whose candidate policies are later evaluated for Pareto optimality. A value in the PDS represents the proportion of the mission dedicated to the exploration phase. The PDS ranges from 1.0 to 0.0 in decrements of 0.1 (therefore 11 values), forming the sequence:

$$PDS_{q}=1.0-0.1q,\;q\in \left\{0,1,2,\ldots ,10\right\}.$$

Here, *q* represents the step index, ensuring a uniform progression from a mission of pure exploration ($PDS_{1}=1.0$) to pure cleaning ($PDS_{0}=0.0$). For instance, a $PDS_{q}$ of 0.3 (q=7) means that 30% of the mission corresponds to exploration (all agents executing exploration behavior), followed by 70% cleaning. During training, the network is periodically evaluated under each PDS configuration. If a newly trained policy surpasses the current best for a specific PDS and objective, it replaces the previous one, ensuring that only the most optimal policies are retained for Pareto evaluation. Then, for each selected PDS value, three distinct policies are stored as Pareto candidates every 500 training episodes: (1) Best Cleaning Policy: The policy achieving the highest cleaning performance within that PDS. (2) Best Exploration Policy: The policy attaining the greatest exploration efficiency within that PDS. (3) Final Trained Policy: The policy obtained at the end of training, which may exhibit improved generalization to unseen scenarios due to prolonged optimization.

Once all candidate policies are trained, Pareto optimality is determined through post-training evaluation. Each policy generated from the Phase Duration Set (PDS) is tested on an independent evaluation set consisting of 200 randomized episodes per environment. During this stage, the average PTC and PMV metrics are measured for each policy. These averaged results are used to determine Pareto optimality and construct the final Fixed-Phase Pareto Set with ours.

**Figure 6:**
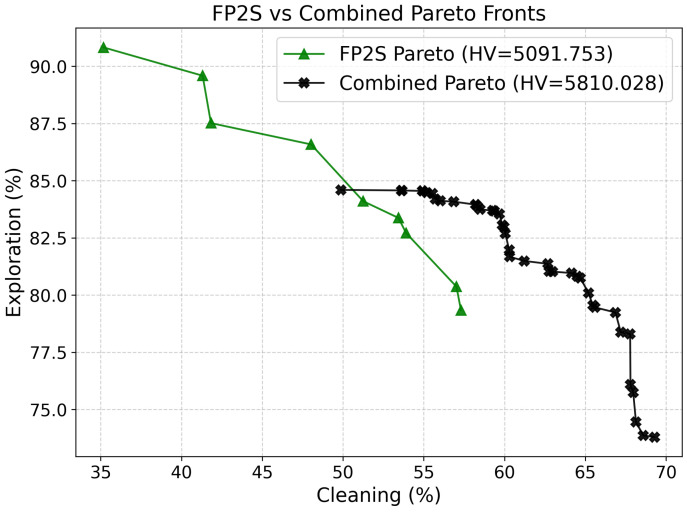
Illustration of the combined pareto front and the FP2S pareto front.

Table 4 reports the performance of the FP2S policies across different fixed exploration-to-cleaning phase ratios (*PDS*). Figure 6 presents a comparative analysis between the Pareto Front obtained with our proposed Combined approach and the FP2S baseline from the literature. The front generated by FP2S exhibits a smaller number of Pareto-optimal solutions, concentrated primarily in regions with high exploration but limited cleaning performance. This scarcity of solutions results in a sparser and less continuous front, which ultimately reduces the achievable hypervolume and diversity. In contrast, the Combined Pareto Front (black crosses) demonstrates a much denser and more evenly distributed set of non-dominated policies, extending further along both objective axes. This indicates that our method achieves a broader and more balanced coverage of the trade-off space between exploration and cleaning.

As detailed in Table 2, our approach achieves a significantly higher hypervolume value (5810.03 compared to 5091.75). The improved hypervolume indicates that the Combined Pareto Front dominates a larger portion of the objective space, offering more favorable trade-offs between exploration efficiency and cleaning effectiveness. Furthermore, the Spacing metric decreases substantially (0.70 versus 2.14), showing that the solutions in our front are more uniformly distributed, providing smoother transitions between neighboring policies. This uniformity is desirable for multi-objective decision-making, as it allows for finer granularity when selecting operating points according to specific mission preferences.

The extension metric $M_{3}$ remains comparable between both methods, although our Combined approach achieves similar coverage with a substantially larger number of points (40 versus 9). This richer set of solutions improves the interpretability and applicability of the Pareto front, giving mission planners a broader range of policies to select from based on operational priorities or environmental conditions. It is worth noting that the FP2S front’s lower diversity is primarily due to its reliance on fixed-duration phase transitions, which limits the variability of exploration–cleaning balance during training. By contrast, our approach combines multiple scalarization strategies of different natures—linear, nonlinear, and exponential—allowing the discovery of policies across a wider spectrum of trade-offs.

Overall, the results clearly demonstrate that the proposed Combined Pareto Front not only surpasses the FP2S baseline in terms of quantitative Pareto metrics but also provides greater adaptability, finer policy resolution, and a more comprehensive representation of the underlying exploration–cleaning dynamics. This enhanced front serves as a more informative and flexible decision-making tool for real-world multi-objective environmental cleanup missions.

**Figure 7:**
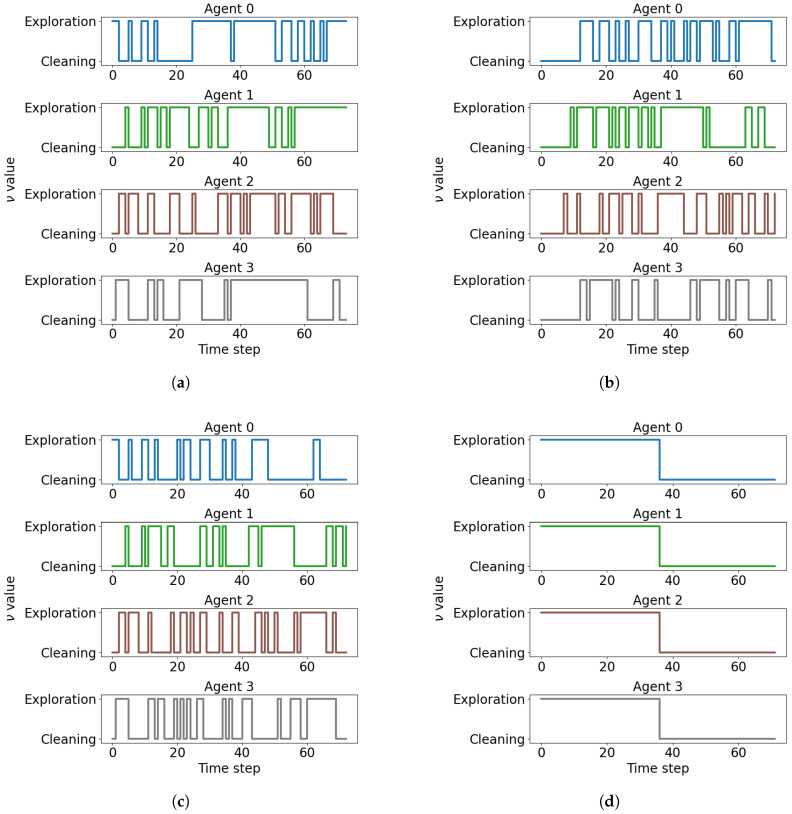
Evolution of ν (task allocation) for each of the four agents across a single representative episode under different scalarization methods and weighting preferences, including an FP2S example. Each subplot shows instantaneous task selections. (**a**) EWC ($w_{1}=0.991$, $w_{2}=0.009$) prioritizes cleaning, (**b**) WPOP ($w_{1}=0.676$, $w_{2}=0.324$) balances both objectives, (**c**) WS ($w_{1}=0.162$, $w_{2}=0.838$) emphasizes exploration, and (**d**) example of FP2S with $PDS_{q}=0.5$, illustrating its characteristic switching pattern. All weight values correspond to Pareto-optimal solutions from the combined front.

Figure 7 illustrates the evolution of task allocations across the four agents for three representative scalarization methods, as well as the evolution produced by FP2S for a given PDS. Each line indicates the instantaneous task selected at each time step (ν=1 for exploration, ν=0 for cleaning). As expected, the EWC configuration (Figure 7a), which heavily prioritizes cleaning ($w_{1}=0.991$, $w_{2}=0.009$), produces trajectories dominated by cleaning action selections, reflecting a predominance of cleaning behavior. Conversely, the WS configuration (Figure 7c), which emphasizes exploration ($w_{1}=0.162$, $w_{2}=0.838$), results in substantially fewer cleaning action selections, consistent with exploratory dominance. The WPOP configuration (Figure 7b) lies between these two extremes, producing a balanced alternation between exploration and cleaning phases. Finally, Figure 7d illustrates FP2S, which—as opposed to the adaptive DRL-based policies—applies a predefined task schedule shared synchronously across all agents, and therefore does not constitute an adaptive policy-switching mechanism. However, the temporal evolution of ν within each episode reveals that the agents’ task decisions are not trivially determined by the scalarization weights. Even in strongly biased configurations, agents exhibit frequent task switches and non-monotonic ν trajectories. This behavior can be attributed to the strong interdependence between exploration and cleaning: an agent’s optimal task choice depends not only on its local context but also on the collective state of the environment and the complementary actions of other agents. Consequently, the system demonstrates emergent coordination, where task allocation dynamically adapts to environmental and inter-agent conditions.

**Adaptation to Different Scales:**

**Figure 8:**
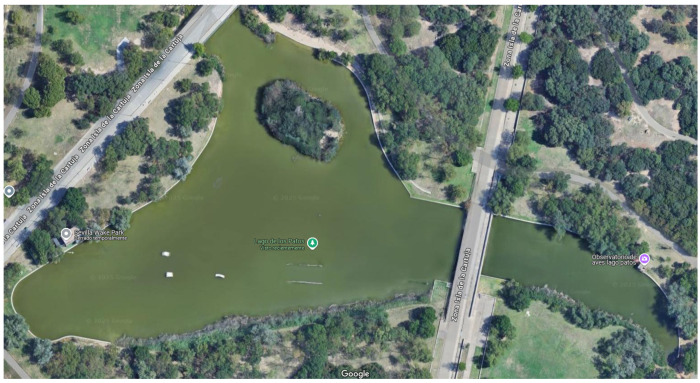
Satellite view of the Alamillo Lake.

**Figure 9:**
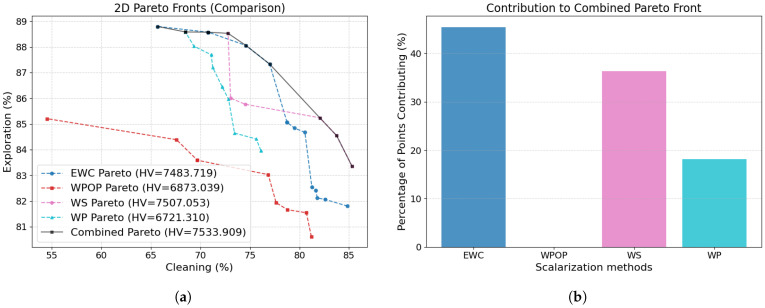
Results of the scalarization methods in Alamillo Lake scenario. (**a**) Pareto fronts obtained using each scalarization method across 100 different weight configurations, along with the resulting combined Pareto front that aggregates all non-dominated solutions. (**b**) Contribution of each scalarization method to the combined Pareto front.

To evaluate the framework’s capacity for generalisation across environments of varying scales and structural complexity, additional experiments were conducted on the Alamillo Lake map (see Figure 8). This map contains 563 navigable nodes, constrained corridors, and internal obstacles. This environment contrasts with Malaga Port, which has 705 navigable nodes (approximately 25% more) and a more open layout. Despite these differences in size and topology, the proposed coordination framework performs consistently (as shown in Figure 9 and Table 5), generating well-formed Pareto fronts in both environments without any environment-specific parameter tuning. Crucially, execution time per decision step remains consistent across environments because task allocation and policy evaluation depend on fleet size rather than map scale. This confirms that the proposed method maintains real-time feasibility when transitioning to structurally different environments. Notably, the Alamillo Lake environment achieves higher overall performance than Malaga Port. This can be attributed to its smaller number of navigable cells, which enables faster spatial coverage under the same step budget. Accelerated exploration increases the likelihood of visiting cells containing trash earlier, which improves the percentage of trash collected directly, since trash is removed immediately upon cell visitation.

#### 3.3.2. Limitations

**Figure 10:**
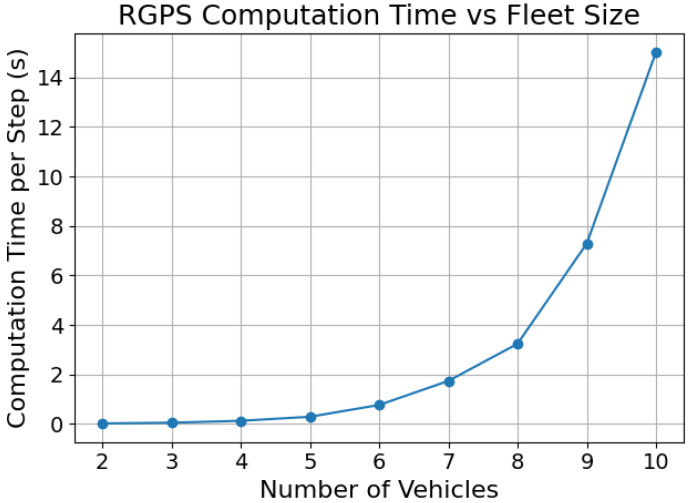
Illustration of the exponential growth in computation time as fleet size increases.

While the proposed RGPS framework demonstrates significant improvements over fixed-phase approaches in multi-agent ASV cleanup operations, it is important to acknowledge several limitations that constrain its current applicability and suggest directions for future research. The most significant limitation of the RGPS mechanism is its exponential computational complexity with respect to fleet size. At each decision step, RGPS evaluates all possible task allocations across the fleet, requiring $2^{N}$ evaluations for *N* agents. While this exhaustive search guarantees optimal task allocation given the current policies, it becomes computationally intractable for large fleets. Figure 10 illustrates the exponential growth in computation time as fleet size increases. Each evaluation step takes approximately 0.13 s on our hardware for *N* = 4, resulting in negligible overhead during mission execution. However, extrapolation suggests that for N≥10, real-time execution would become challenging with the current implementation. Our method is designed for and evaluated on small to medium-sized ASV fleets, which are representative of current harbor, coastal, and lake cleanup deployments. For such fleet sizes, the computational overhead remains manageable and compatible with typical control loop frequencies. However, scaling to larger fleets would require algorithmic modifications.

## 4. Conclusions

This work introduces a multi-agent deep reinforcement learning framework for autonomous plastic cleanup using fleets of ASVs. The proposed system formulates the problem as a POMG, decoupling the mission into two complementary tasks: exploration, to map and locate waste, and cleaning, to collect it efficiently. Both tasks share navigation capabilities but involve conflicting objectives, motivating a multi-objective optimization approach. A shared two-headed Deep Q-Network, with one head dedicated to exploration and the other to cleaning, allows all agents to benefit from parameter sharing and experience aggregation.

To enhance adaptive decision-making, we introduce the Reward-Greedy Policy Selection (RGPS) mechanism, inspired by the principles of adaptive submodularity [23,24]. RGPS evaluates all feasible task allocations across the fleet at each time step and greedily selects the configuration that maximizes a scalarized reward. This explicit optimization accounts for interdependencies between agents’ actions and enables per-agent, per-timestep task allocation without additional learning or training. By leveraging separate policies for exploration and cleaning, RGPS supports coordinated yet specialized behavior, making it well suited to the dynamic and stochastic nature of aquatic environments, particularly in small to medium-sized ASV fleets.

The proposed approach generates a well-distributed Pareto front, revealing diverse strategies across the exploration–cleaning spectrum. Compared to the Fixed-Phase Pareto Set (FP2S) baseline from the literature, our framework achieves a 14% improvement in hypervolume and a 300% increase in uniformity, confirming superior Pareto coverage and policy diversity. Moreover, the system exhibits adaptive behavior across structurally different environments, automatically adjusting phase durations to maximize mission efficiency. The proposed framework is not limited to simulation and is currently being integrated into a real-world mission planning, navigation, and control stack for autonomous surface vehicles. Although real-world deployment poses challenges such as environmental disturbances and communication instability, the methodology remains practically transferable, and ongoing work focuses on experimental validation on physical ASV platforms.

Future work will focus on developing more computationally efficient approaches for constructing Pareto fronts, such as Multi-Objective Evolutionary Algorithms (MOEAs), which can evolve a diverse set of trade-off solutions over successive generations. Another promising direction is to incorporate mission duration as an additional objective, thereby extending the optimization to a three-dimensional Pareto front that captures the interplay between exploration, cleaning, and time efficiency. Moreover, adaptive mechanisms capable of dynamically adjusting the exploration–cleaning balance based on real-time environmental feedback could be explored. This adaptability could be realized through Hierarchical Reinforcement Learning (HRL), where a high-level policy governs the switching between tasks, while low-level controllers handle task-specific decision-making. Additionally, while our current study assumes reliable communication, we recognize that real-world conditions can be more challenging. Future work will evaluate algorithm performance under intermittent or limited-bandwidth communication conditions. Furthermore, future work will prioritize systematic comparisons with state-of-the-art MO-MADRL approaches as reproducibility practices and benchmarking protocols in the field become more standardized.

## Figures and Tables

**Table 1:** Simulation environment, learning, and scalarization hyperparameters.

Category	Parameter	Value
Scalarization	Weighted Product exponent (α)	3
	Exponential Weighted Criterion (*p*)	1
Environment	Number of ASVs (*N*)	4
	Detection radius (ρ)	2 nodes
	Distance budget	200 units
	Movement length	1 node
Training	Replay buffer size	$1\times 10^{6}$
	Batch size	128
	Learning rate (LR)	$1\times 10^{-4}$
	Target network hard update	2000 steps
	ϵ-greedy interval	[1,0.05]
	ϵ threshold	50%
	Discount factor (γ)	0.99

**Table 2:** Summary of Pareto front quality metrics for each reward scalarization and the combined front.

Scalarization	Hypervolume	M3	Spacing	Nº of Points
WP	5400.8738	5.0406	0.9193	21
WS	5779.8875	5.4511	1.4368	19
WPOP	5750.2380	5.0639	0.6876	21
EWC	5706.1609	5.2529	1.0877	23
Combined	5810.0283	5.4977	0.7035	40
Literature	5091.7528	5.7991	2.1422	9

**Table 3:** Wilcoxon signed-rank test results between Pareto fronts for each reward configuration. Significant (p<0.05) differences are highlighted in bold.

Comparison	Cleaning (*p*-Value)	Exploration (*p*-Value)
WP vs. WS	0.2753	0.0955
WP vs. WPOP	**0.0239**	0.5392
WP vs. EWC	0.6578	**0.0022**
WS vs. WPOP	0.2935	0.1956
WS vs. EWC	0.3321	0.1956
WPOP vs. EWC	0.1111	0.1193

**Table 4:** FP2S candidate Pareto-optimal policies evaluated over 200 episodes. The policy labels follow the notation Policy (Type @ PDS), where Type ∈{BestExploration,BestCleaning,FinalTrained} policies, and PDS denotes the fixed exploration duration (e.g., 0.7 represents a mission with 70% exploration time followed by cleaning).

Policy (Type @ PDS)	Cleaning (%)	Exploration (%)
Best Exploration @ 1.0	35.2	90.8
Best Exploration @ 0.9	41.3	89.6
Best Cleaning @ 0.9	41.8	87.5
Best Exploration @ 0.8	48.0	86.6
Best Exploration @ 0.7	51.3	84.1
Best Cleaning @ 0.7	53.4	83.4
Final Trained @ 0.7	53.9	82.7
Best Exploration @ 0.6	57.0	80.4
Best Cleaning @ 0.6	57.3	79.3

**Table 5:** Summary of Pareto front quality metrics for each reward scalarization method and the combined Pareto front on the Alamillo Lake environment.

Scalarization	Hypervolume	M3	Spacing	Nº of Points
EWC	7483.7189	5.1127	1.5835	12
WPOP	6873.0391	5.5886	4.4323	8
WS	7507.0533	4.2034	2.5124	6
WP	6721.3104	3.4942	0.7545	9
Combined	7533.9085	5.0035	1.5274	11

## Data Availability

The original contributions presented in this study are included in the article. Further inquiries can be directed to the corresponding authors.

## Abbreviations

The following abbreviations are used in this manuscript:

POMG	Partially Observable Markov Game
ASV	Autonomous Surface Vehicle
ML	Marine Litter
DRL	Deep Reinforcement Learning
DQL	Deep Q-Learning
DQN	Deep Q-Network
MADRL	Multi-Agent Deep Reinforcement Learning
MDQN	Multi-Agent Deep Q-Network
MT-MADRL	Multi-Task Multi-Agent Deep Reinforcement Learning
CNN	Convolutional Neural Network
C-DQL	Censoring Deep Q-Learning
MDP	Markov Decision Process
RGPS	Reward Greedy Policy Selection
WS	Weighted Sum
WP	Weighted Power
WPOP	Weighted Product of Powers
PTC	Percentage of Trash Cleaned
PMV	Percentage of the Map Visited
HV	Hypervolume
FP2S	Fixed-Phase Pareto Set
PDS	Phase Duration Set
MOEAs	Multi-Objective Evolutionary Algorithms
HRL	Hierarchical Reinforcement Learning
